# Susceptibility to organophosphate insecticides in *Aedes aegypti* (Diptera: Culicidae) from northern Colombia and associated resistance mechanisms

**DOI:** 10.1186/s13071-024-06624-8

**Published:** 2025-01-14

**Authors:** María Claudia Atencia-Pineda, Javier García-Leal, Diana Diaz-Ortiz, Paula Pareja-Loaiza, Lisandro Pacheco-Lugo, Richard Hoyos-López, Alfonso Calderón-Rangel, Pedro Fragozo-Castilla, Selene M. Gutiérrez-Rodríguez, Adriana E. Flores, Ronald Maestre-Serrano

**Affiliations:** 1 Doctorado en Microbiología y Salud Tropical, Facultad de Medicina Veterinaria y Zootecnia, Universidad de Córdoba, Montería, Colombia; 2 Facultad de Ciencias Básicas y Biomédicas, Centro de Investigación en Ciencias de la Vida (CICV), Universidad Simón Bolívar, Barranquilla, Colombia; 3 Facultad de Ciencias de la Salud, Centro de Investigación en Ciencias de la Vida (CICV), Universidad Simón Bolívar, Barranquilla, Colombia; 4 Instituto de Investigaciones Biológicas del Trópico (IIBT), Universidad de Córdoba, Montería, Colombia; 5 Grupo de Investigación Parasitología Agroecología Milenio, Universidad Popular del Cesar, Valledupar, Colombia; 6 Facultad de Ciencias Biológicas, Universidad Autónoma de Nuevo León, San Nicolás de los Garzas, Mexico

**Keywords:** Organophosphates, *Aedes aegypti*, Susceptibility screening, Metabolic resistance, Córdoba

## Abstract

**Background:**

*Aedes aegypti* is the primary vector of dengue, chikungunya, and Zika viruses in Colombia. Various insecticides, including pyrethroid, organophosphate, and carbamate insecticides; growth regulators; and biological insecticides, such as *Bacillus thuringiensis* var. *israelensis*, have been used to control *Ae. aegypti* populations. However, organophosphates such as malathion, pirimiphos-methyl, and temephos have been used over the last decade owing to the high resistance to pyrethroids.

**Methods:**

This study assessed the susceptibility to organophosphates in 14 *Ae. aegypti* populations from the Córdoba department in northern Colombia. Moreover, possible resistance mechanisms were investigated by determining the activity levels of α-esterases, β-esterases, mixed function oxidases (MFOs), glutathione S-transferases (GSTs), and insensitive acetylcholinesterase (iAChE). Additionally, the *Ace-1* gene was sequenced to identify mutations at the target site of action.

**Results:**

The populations were susceptible to temephos and malathion but resistant to fenitrothion, and in three of them, to pirimiphos-methyl. Alterations in the enzyme activity levels of α-esterases and β-esterases, GST, and iAChE were observed among the populations, with high enzyme activity levels of α and β esterases associated with resistance to fenitrothion. No mutations were identified in the *Ace-1* gene.

**Conclusions:**

These findings are highly relevant for vector control programs in the region, as they allow for adjustments in resistance management strategies and improve the effectiveness of interventions against these arboviruses.

**Graphical Abstract:**

**Figure:**
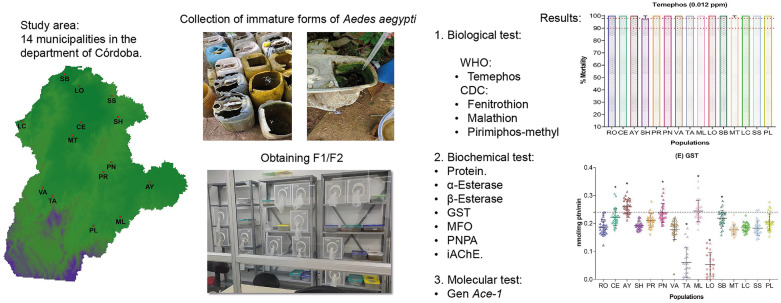

**Supplementary Information:**

The online version contains supplementary material available at 10.1186/s13071-024-06624-8.

## Background

Dengue, chikungunya, and Zika viruses are major public health concerns in Colombia. These viruses are spread by *Aedes aegypti*, which is present in 80% of the country and can be found at altitudes up to 2302 m above sea level [1]. Approximately 328,654 cases of dengue, 4940 cases of severe dengue, and 273 confirmed deaths were reported between 2020 and 2023. During the same period, approximately 303 cases of Zika and 344 cases of chikungunya were confirmed following previous epidemics in the country [2–5].

In the Córdoba department, dengue has exhibited an endemic–epidemic pattern, with 12,261 cases and 13 confirmed deaths between 2020 and 2023 [2–5]. The municipalities with the highest incidence during this period were Montería (3089 cases), Montelíbano (327 cases), Valencia (313 cases), Tierralta (293 cases), Ciénaga de Oro (254 cases), and Cereté (252 cases). Additionally, two cases of chikungunya and four cases of Zika were also reported [6–9].

Surveillance, prevention, and control of these diseases in Colombia are based on the Integrated Management Strategy (EGI, for its Spanish acronym), which aims to mitigate the immediate transmission through integrated vector control, reducing or eliminating the risk factors that facilitate virus transmission between humans and vectors [10]. However, chemical control is still the most widely used strategy to disrupt the transmission of these diseases during outbreaks and epidemics [11].

Since the 1970s, organophosphates such as temephos and fenitrothion have been used for vector control in Colombia. Since the 1980s, malathion has been the most widely used insecticide in the country for controlling dengue outbreaks or epidemics through space spraying [12]. Despite the rotation of organophosphates and pyrethroids for controlling vectors during outbreaks [13], insecticide resistance has been reported in *Ae. aegypti* populations, complicating control efforts and contributing to the increase in disease cases, especially dengue [14–17].

In the Córdoba department, the status of susceptibility to organophosphates in *Ae. aegypti* populations have been evaluated in three municipalities. In 2014, Maestre et al. reported susceptibility to malathion, fenitrothion, and pirimiphos-methyl in Montería; however, in 2018, the Entomology Laboratory of the National Institute of Health (Colombia) reported resistance to fenitrothion in San Bernardo del Viento and to pirimiphos-methyl in Pueblo Nuevo [16].

Given this situation, it is necessary to determine the susceptibility to organophosphates in the municipalities of Córdoba that lack a baseline of susceptibility data and update the information in those that already have it, especially in areas with a high incidence of arboviruses. This will provide valuable data that may be used to improve vector control strategies.

This study aimed to evaluate the susceptibility to organophosphates in *Ae. aegypti* from 14 municipalities from the Córdoba department and identify the associated biochemical and molecular resistance mechanisms.

## Methods

### Study area

**Fig. 1:**
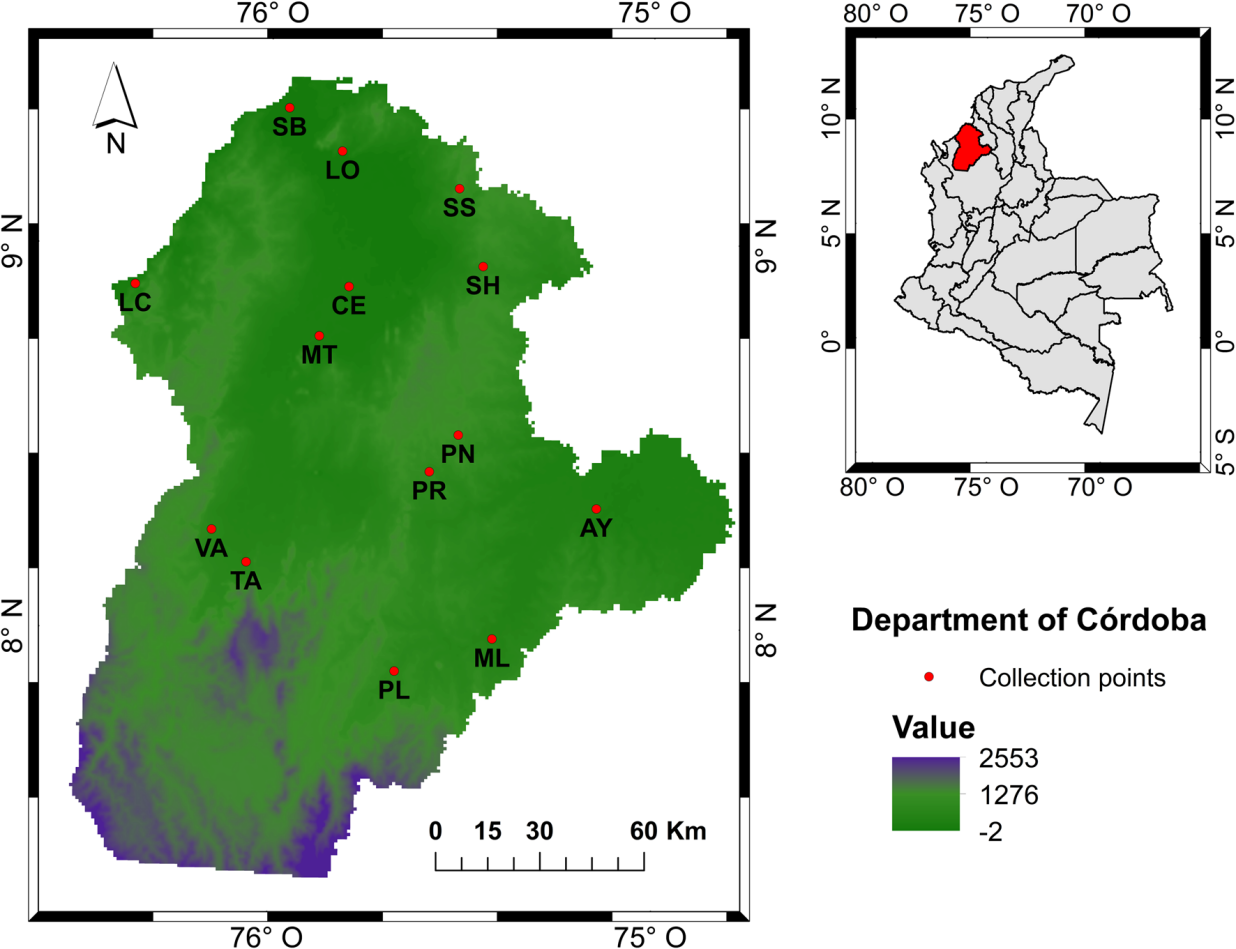
*Ae. aegypti* populations in the Córdoba department, Colombia. *CE* Cereté, *SH* Sahagún, *PR* Planeta Rica, *SB* San Bernardo del Viento, *LO* Lorica, *AY* Ayapel, *ML* Montelíbano, *TA* Tierralta, *PN* Pueblo Nuevo, *VA* Valencia, *MT* Montería, *SS* San Andrés de Sotavento, *LC* Los Córdobas, *PL* Puerto Libertador

The study was conducted in the Córdoba department, located in the Colombian Caribbean region in the northwest of Colombia, and included the following municipalities: Ayapel, Cereté, Lorica, Los Córdobas, Montelíbano, Montería, Planeta Rica, Pueblo Nuevo, Puerto Libertador, Sahagún, San Andrés de Sotavento, San Bernardo del Viento, Tierralta, and Valencia (Fig. 1). The municipalities were selected according to the inclusion criteria on the basis of the number of cases of dengue, chikunguya, and Zika reported from 2015 to 2020.

The housing infestation index in the municipalities under study for the year 2022 was as follows: Ayapel (22.4%) Cereté (36%), Lorica (21%), Los Córdobas (48.7%), Montelíbano (30.6%), Montería (41.3%), Planeta Rica (14.3%), Pueblo Nuevo (54%), Puerto Libertador (18.8%), Sahagún (23.5%), San Andrés de Sotavento (20.8%), San Bernardo del Viento (51.5%), Tierralta (35%), and Valencia (37%) (unpublished data) National Institute of Health.

### Collection and establishment of the parental generation of *Ae. aegypti* from Córdoba

The *Ae. aegypti* populations were obtained between May and December 2022 directly from the field in larval stages from different neighborhoods in the selected municipalities. The neighborhoods were chosen on the basis of recommendations from the municipal health departments, considering their history of high dengue incidence or high vector infestation. Water containers—including plastic tanks, cement tanks, flower vases, bottles, aquatic plants, tires, and unusable items—were inspected in intra- and peri-domiciliary areas of selected households. Samples were collected from 300 to 400 containers per district.

The collected larvae were stored in 5 L plastic tanks and transported to the University of Córdoba and Simón Bolívar University insectaries. The collected mosquitoes were considered as the parental generation, which was used to obtain the F_1_ generation under the following controlled conditions: temperature at 28 ± 2 °C, relative humidity at 60 ± 10%, and a 12 h light:12 h dark photoperiod.

### Bioassays

#### Susceptibility bioassays to temephos in *Ae. aegypti* larvae

Susceptibility to temephos was evaluated according to the parameters and procedures recommended by the World Health Organization (WHO) [18]. Technical grade temephos (purity: 97.3%, Chem Service) was diluted in ethanol to obtain a discriminating concentration (DC) of 0.012 ppm when diluted in distilled water to get a volume of 100 mL. Third-instar larvae of F_1_ generation from the 14 populations and the Rockefeller reference strain were used for the bioassays, and four replicates were performed, each containing 15–25 larvae and a control comprising 1 mL of ethanol diluted in water. Mortality readings were taken 24 h post-exposure.

#### Susceptibility bioassays to organophosphates in *Ae. aegypti* adults

Bioassays for female mosquitoes were conducted with fenitrothion, malathion, and pirimiphos-methyl (purities 98%, 98.6%, and 98.4%, respectively; Chem Service), following the bottle bioassay [19] and the guidelines provided by the Centers for Disease Control and Prevention (CDC) [20].

The bioassays consisted of exposing 15–25 non-blood-fed, 3–5-day-old F_1_ female mosquitoes of each study population and the Rockefeller control strain to the DC of insecticides diluted in absolute ethanol (fenitrothion, 50 µg/bottle; malathion, 50 µg/bottle; pirimiphos-methyl, 75 µg/bottle). Mortality for fenitrothion and malathion was evaluated within the diagnostic time of 30 min, as recommended by the CDC [20], and within the diagnostic time of 45 min for pirimiphos-methyl, as reported by the National Institute of Health of Colombia. Readings were taken every 5 min until the diagnostic time was reached. A control bottle was included, impregnated with 1 mL of absolute ethanol. Correction of mortality was not necessary, as no mortality was observed in the controls.

For populations where resistance at diagnostic dose and time was recorded, the intensity of resistance was determined by conducting new bioassays that involved evaluating doses two (2×), five (5×), or ten (10×) times the DC [21].

Mortality at diagnostic time for each population was classified according to the WHO interpretation criteria [21]. A 98–100% mortality was considered susceptible, 90–97% indicated probable resistance, and <90% mortality indicated resistance. The intensity of resistance results was interpreted according to the WHO guidelines [21].

### Biochemical assays

Biochemical assays were conducted on *Ae. aegypti* females from the parental generation (field collected) that were less than 24 h old; the parental generation was used because the heterogeneous nature of this population may present results closer to field reality. A total of 70 unfed females from each population were stored at −80 °C until further processing. Enzyme activities of α-esterases and β-esterases, glutathione S-transferases (GSTs), mixed function oxidases (MFOs), acetylcholinesterase (AChE), and insensitive acetylcholinesterase (iAChE) were evaluated; 30 mosquitoes from each population and the Rockefeller reference strain were analyzed. Each mosquito was homogenized in 200 μL of 0.05 M potassium phosphate (KPO_4_) buffer, pH 7.2, and the homogenates were brought to a final volume of 2000 μL using the same buffer. Samples were divided into 100 μL aliquots and analyzed in triplicate in 96-well plates for each enzyme, following the methodology of Brogdon [22] and Brogdon and Barber [23].

For the pNPA-esterase enzymatic activity, 30 females from each population were macerated in 30 μL of ultrapure water for 5–10 s, with an additional 270 μL of ultrapure water added to reach a final volume of 300 μL. Samples were centrifuged at 12,000 rpm for 60 s and distributed in 10 μL aliquots in duplicate on 96-well microplates, following the methodology of Valle et al. [24]. In both procedures, the total protein concentration of each mosquito was determined to adjust for body size differences [25]. Positive and negative controls were included for MFO and esterases, using the same volume of homogenate of the corresponding tests. For α- and β-esterases, solutions of α- and β-naphthyl acetate were used, respectively, and cytochrome-c solution served as the positive control for MFO. The KPO_4_ buffer served as a negative control. Absorbance levels were measured using an Enzyme-Linked ImmunoSorbent Assay (ELISA) plate reader (Multiskan TM, Thermo Fisher Scientific^®^) at specific wavelengths for each enzyme, and the mean absorbance was calculated on the basis of the replicates. Replicates with a variation coefficient higher than 0.2 were discarded to avoid differences caused by manual errors [26]. Absorbance values obtained from mosquito replicates were corrected relative to the volume of mosquito homogenate, enzyme activity unit, and total protein content of each mosquito. The mean enzyme activity of each field population was compared with the Rockefeller strain using the nonparametric Kruskal–Wallis test (*α* = 0.05). The 99th percentile of the Rockefeller strain was used as a threshold to determine the level of enzymatic alteration. The activity was then classified as unaltered (< 15%), incipiently altered (15–50%), and altered (> 50%) [27].

To assess the relationship between enzymatic activity and the observed resistance to fenitrothion and pirimiphos-methyl, a Pearson correlation analysis was conducted (*a* = 0.05). The analyses were performed in GraphPad Prism version 9.

### Identification of mutations in the *Ace-1* gene

DNA was extracted from 40 mosquitoes from each population of the parental generation (field collected) using the Quanta Biosciences Extracta TM kit. Mosquitoes were placed individually in 0.2 mL sterile tubes containing 25 µL of extraction buffer and incubated at 95 °C for 30 min in a thermal cycler. At the end of incubation, 25 µL of stabilization buffer was added. DNA was quantified using a NanoDrop 2000/2000c (Thermo Fisher Scientific) spectrophotometer.

A conventional polymerase chain reaction (PCR) was performed to amplify a region of approximately 500 bp of the gene encoding acetylcholinesterase (AChE) using the primers designed by Muthusamy and Shivakumar [28]. The primer sequences are forward: 5′-CGATAACGAATGGGGAACG-3′ and reverse: 5′-TCAGAGGCTCACCGAACACA-3′. The final reaction volume was 30 µL, composed of 0.6 µL of each primer (10 µM), 15 µL of Master Mix GoTaq G2 Colorless (Promega), 10.8 µL of molecular biology grade H_2_O, and 3.0 µL of DNA (100–500 ng/µL). The amplification conditions were as follows: initial denaturation at 95 °C for 3 min, followed by 35 cycles of 95 °C for 30 s, 57 °C for 1 min, and 72 °C for 2 min; and a final extension at 72 °C for 7 min. The products were visualized on 1.5% agarose gel and sent to Macrogen Inc. (Seoul, South Korea) for sequencing.

The obtained DNA sequences were edited using Mega v10 software [29], and mutations and allelic variants were determined by sequence analysis. The BLAST tool was used to verify whether the amplified fragment corresponded to the *Ace-1* gene of *Ae. aegypti*, deposited in GenBank under accession number BK006052.1, and to identify the homologous positions of the initial and final nucleotides according to the mRNA sequence deposited in GenBank under accession number EF209048.1.

The sequences obtained were deposited in GenBank under accession numbers PP763441, PP763442, and PP763443.

## Results

### Bioassays

All the *Ae. aegypti* populations included in this study showed susceptibility to temephos (mortality rates between 98% and 100%) and malathion (Table 1). However, resistance to fenitrothion was observed in all 14 populations. The population from Planeta Rica had the lowest mortality rate (2.9%), whereas the population from Los Córdobas showed the highest mortality rate (72.3%). Additionally, the populations from Montelíbano and Lorica showed resistance to pirimiphos-methyl, with mortality rates of 75.4% and 89.3%, respectively. In the Sahagún population, possible resistance was observed with a mortality rate of 95.6% (Table 1) “Additional File 1: Dataset S1”.

**Table 1:** Frequency and intensity of resistance of female *Ae. aegypti* mosquitoes from different municipalities of Córdoba, Colombia, exposed to the 1× and 2×, 5×, or 10× discriminating concentrations of organophosphate adulticides

Population	Organophosphates															
	Fenitrothion								Pirimiphos-methyl						Malathion	
	1× DC		2× DC		5× DC		10× DC		1× DC		2× DC		5× DC		1× DC	
	DC: 50 μg/bottle; DT: 30 min		DC: 100 μg/bottle; DT: 30 min		DC: 250 μg/bottle; DT: 30 min		DC: 500 μg/bottle; DT: 30 min		DC: 75 μg/bottle; DT: 45 min		DC: 150 μg/bottle; DT: 45 min		DC: 375 μg/bottle; DT: 45 min		DC: 50 μg/bottle; DT: 30 min	
	*n*	%	*n*	%	*n*	%	*n*	%	*n*	%	*n*	%	*n*	%	*n*	%
Cereté	71	9.9	78	14.2	83	34.6	73	69.3	93	100	–	–	–	–	83	100
Ayapel	71	7.6	70	18.4	69	100	–	–	80	100	–	–	–	–	69	100
Sahagún	78	22.9	80	74.4	90	98.9	–	–	66	95.6	76	98.6	–	–	73	100
Planeta Rica	79	2.9	80	6.51	85	50.4	82	95.2	77	100	–	–	–	–	78	100
Pueblo Nuevo	83	20.1	84	42.8	69	52.3	79	90.9	79	100	–	–	–	–	72	100
Valencia	79	40.9	82	65.9	79	83.6	82	95.2	85	100	–	–	–	–	72	100
Tierralta	72	3.4	68	12.7	86	46.6	78	58.8	71	98.6	–	–	–	–	72	100
Montelíbano	69	11.5	74	17.6	71	72.9	71	98.6	81	75.4	70	95.7	77	100	83	100
Lorica	74	13.4	82	18.3	78	52.6	93	66.6	86	89.3	80	100	–	–	72	100
San Bernardo del Viento	76	12.5	74	22.9	90	31.3	72	47.4	75	98.7	–	–	–	–	73	100
Montería	73	12.6	75	51.2	84	98.9	–	–	78	100	–	–	–	–	67	100
Los Córdobas	79	72.3	77	77.7	73	93.6	71	95.5	91	98.9	–	–	–	–	73	100
San Andrés de Sotavento	83	10.8	73	20.5	78	29.4	70	46.5	73	100	–	–	–	–	72	100
Puerto Libertador	72	6.9	88	18.2	70	38.6	74	40.7	67	100	–	–	–	–	78	98.7

*n* number of females bioassayed, *%* mortality rate, *DC* discriminating concentration, *DT* diagnostic time

When evaluating the intensity of resistance to fenitrothion, the populations from Montería, Sahagún, and Ayapel showed low levels of resistance; Montelíbano showed moderate intensity of resistance; and the populations from Cereté, Planeta Rica, Pueblo Nuevo, Valencia, Tierralta, Lorica, San Bernardo del Viento, Los Córdobas, San Andrés de Sotavento, and Puerto Libertador exhibited high intensity of resistance. Further, the intensity of resistance to pirimiphos-methyl in the initially resistant populations was classified as low (Table 1).

### Biochemical assays

The 99th percentile values determined for the enzymes in Rockefeller strain were as follows: α-esterase (3.50 nmol/mg ptn/min), β-esterase (15.48 nmol/mg ptn/min), pNPA-esterase (28.40 ∆Abs/mg ptn/min), MFOs (54.91 nmol cit/mg ptn), GST (0.24 nmol/mg ptn/min), and iAChE (87.81% inhibition).

**Fig. 2:**
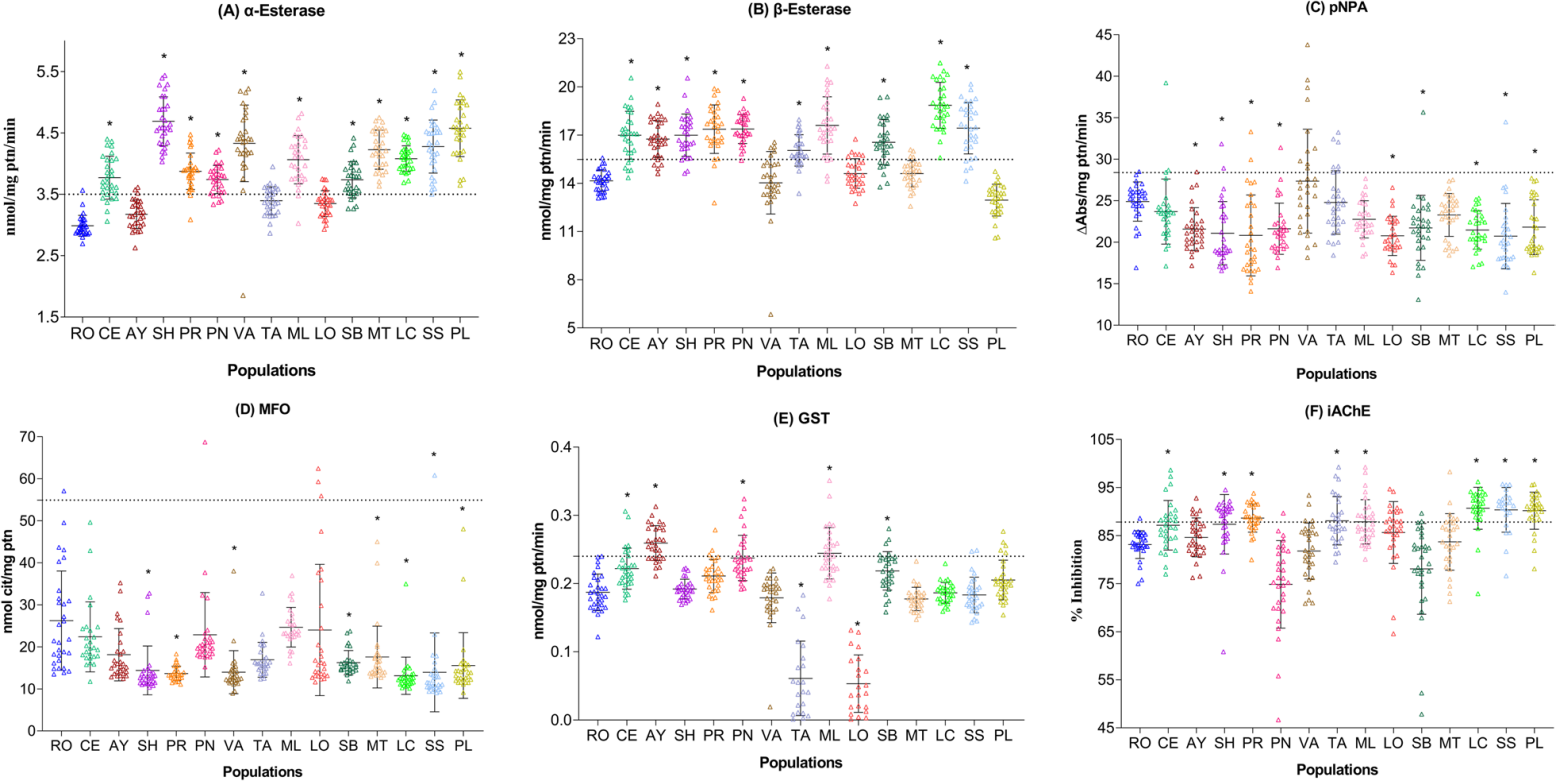
Enzyme activity of each population of *Ae. aegypti* females from different municipalities of Córdoba, Colombia; **A** α-esterases, **B** β-esterases, **C** pNPA- esterases, **D** mixed function oxidases (MFOs), **E** glutathione-*S*-transferase (GST), and **F** insensitive acetylcholinesterase (iAChE). *RO* Rockefeller, *CE* Cereté, *AY* Ayapel, *SH* Sahagún, *PR* Planeta Rica, *PN* Pueblo Nuevo, *VA* Valencia, *TA* Tierralta, *ML* Montelíbano, *LO* Lorica, *SB* San Bernardo del Viento, *MT* Montería, *LC* Los Córdobas, *SS* San Andrés de Sotavento, *PL* Puerto Libertador. The dotted line indicates the 99th percentile determined in the Rockefeller reference strain. Asterisks denote the populations that showed significant differences in enzymatic alteration levels compared with the Rockefeller reference strain (*P* < 0.0001)

On the basis of the classification criteria by Montella et al. (2007), α-esterase activity levels were found to be altered in the populations from Montería (100%), Los Córdobas (100%), Puerto Libertador (100%), Sahagún (100%), San Andrés de Sotavento (100%), Valencia (97%), Montelíbano (93%), Planeta Rica (93%), San Bernardo del Viento (83%), Cereté (80%), and Pueblo Nuevo (80%); incipiently altered in the populations from Tierralta (37%) and Lorica (20%); and unaltered in the population from Ayapel (10%) (Fig. 2A).

Similarly, β-esterase activity levels were altered in the populations from Los Córdobas (100%), Pueblo Nuevo (97%), Planeta Rica (93%), Montelíbano (90%), Sahagún (90%), San Andrés de Sotavento (87%), Ayapel (83%), San Bernardo del Viento (77%), Tierralta (77%), and Cereté (53%); and incipiently altered in the populations from Lorica (20%) and Valencia (17%) (Fig. 2B). pNPA-esterase activity levels were incipiently altered in the populations from Valencia (33%) and Tierralta (23%), whereas the other populations did not exhibit enzymatic alteration (Fig. 2C). The activity of MFOs remained unaltered in all the study populations (Fig. 2D). GST activity levels were altered in the population from Ayapel (70%) and moderately and incipiently altered in those from Montelíbano (47%), Pueblo Nuevo (30%), Cereté (23%), Puerto Libertador, and San Bernardo del Viento (17%) (Fig. 2E). iAChE activity was found to be altered in the populations from Los Córdobas (90%), Puerto Libertador (86%), Planeta Rica (63%), Sahagún (60%), and San Andrés de Sotavento (77%); and moderately and incipiently altered in all other populations, except for San Bernardo del Viento, Valencia, and Pueblo Nuevo (Fig. 2F).

Significant enzymatic activity differences were observed between most field populations and the Rockefeller reference strain. The Kruskal–Wallis test showed the following statistical values for each enzyme evaluated: α-esterase (*H* = 319.8), β-esterase (*H* = 287.8), pNPA-esterase (*H* = 97.1), MFOs (*H* = 206.9), GST (*H* = 278), iAChE (*H* = 184.3), *df* = 14, (*P* < 0.0001).

No significant correlation was found between enzymatic activity and resistance to fenitrothion and pirimiphos-methyl in the studied populations. The Pearson correlation analysis results were as follows: for fenitrothion–α-esterase, *r* = 0.26, *P* = 0.36; fenitrothion–β-esterase, *r* = 0.27, *P* = 0.35; fenitrothion–GST, *r* = 0.07, *P* = 0.79; fenitrothion–iAChE, *r* = 0.038, *P* = 0.89; pirimiphos-methyl–α-esterase, *r* = 0.22, *P* = 0.85; pirimiphos-methyl–β-esterase, *r* = −0.39, *P* = 0.74; pirimiphos-methyl–GST, *r* = −0.21, *P* = 0.86; pirimiphos-methyl–iAChE, *r* = −0.41, *P* = 0.73.

### Mutations in *Ace-1* gene

A 500 bp fragment of the *Ace-1* gene, corresponding to nucleotides 1690–2189 of the cDNA sequence of the messenger RNA (GenBank accession number EF209048.1) was amplified from 40 individuals from each of the study populations.

Two synonymous mutations were identified, one at position 1797 (GAC to GAT) and another at position 1983 (ACA to ACT). No polymorphic amino acid sites were found within these sequences.

Of the 560 samples analyzed, three genetic variants were identified in the partial sequences of the *Ace-1* gene. These sequences have been deposited in the GenBank database under accession numbers PP76344, PP76345, and PP76346.

## Discussion

In Colombia, at the beginning of the 1970s, temephos applications were carried out every 2 months per year, later the use of temephos was restricted for use only in cases of outbreaks. In this study, all the evaluated *Ae. aegypti* populations were susceptible to temephos, which is consistent with the findings of Maestre-Serrano et al. [26] and Pareja-Loaiza [17], who reported susceptibility to this larvicide in the *Ae. aegypti* population from Montería. Previously, in 2011, resistance to temephos had been reported in populations from Pueblo Nuevo and Planeta Rica on the basis of monitoring conducted by the National Network of Surveillance of Insecticide Resistance of the National Institute of Health (unpublished data). Following these findings, the growth regulator diflubenzuron (Dimilin) has been used since 2012 for larval control in the department of Córdoba. Although susceptibility observed in the populations from Pueblo Nuevo and Planeta Rica differs from the results reported in 2011, this difference may be attributed to the reversal of resistance to this larvicide, previously documented for this species in Colombia [26] and in other countries in the region [30, 31]. Lima et al. [32] found that the reduction of resistance to temephos in the field can be slow and could take approximately 7 years. However, Prieto et al. [33] demonstrated that resistance can be reversed within 3 years upon suspension of temephos application. Meanwhile, Bisset et al. [31] observed that the intensity of resistance to temephos can revert in just six generations.

On the basis of these studies, it can be inferred that the susceptibility observed in the Pueblo Nuevo and Planeta Rica populations is owing to the reversal of resistance resulting from the non-use of this larvicide for over 10 years. Similar results of susceptibility to temephos have been observed in *Ae. aegypti* populations from the Caribbean region of Colombia, including Barranquilla (Atlántico), Cartagena (Bolívar), Ciénaga (Magdalena), Montería (Córdoba), San Juan (La Guajira), Sincelejo (Sucre), Valledupar (Cesar), Chiriguaná (Cesar), and Juan de Acosta (Atlántico) [17, 26], as well as in various sites in the department of La Guajira [34]. In contrast, resistance has been reported in *Ae. aegypti* from the municipalities of Malambo, Baranoa, Sabanagrande, and Puerto Colombia (Atlántico department) [26, 35], in Cúcuta (North Santander) [36], and from the departments of Cauca, Nariño, Huila, Valle del Cauca [37], Cundinamarca, Guaviare, Meta, and Santander [12].

In this study, all *Ae. aegypti* populations were also susceptible to malathion. Although this organophosphate is the most widely used in Colombia for vector control during outbreaks or epidemics, particularly of dengue [37], and has been used in the department of Córdoba since 2018 (unpublished data), reports of resistance in the country are scarce [38]. Similar results of susceptibility to malathion have been reported in Antioquia, Chocó, Putumayo [39], Cauca, Huila, Nariño, Valle del Cauca [37], Atlántico, Sucre, Cesar, Magdalena, La Guajira [16, 17, 26, 34, 35], and in the municipality of Montería in the department of Córdoba [17, 26]. This result indicates that it is feasible to continue using malathion for vector control in these populations. However, continuous monitoring should be performed to detect any variations in susceptibility levels to the insecticide.

Resistance was observed for pirimiphos-methyl in populations from Montelíbano and Lorica. Pirimiphos-methyl was used for vector control in the department of Córdoba from 2016 to 2017. In 2018, the National Network of Surveillance of Insecticide Resistance reported resistance in *Ae. aegypti* from Pueblo Nuevo [16]. Current results show that *Ae. aegypti* from this location is now susceptible to this insecticide, suggesting a reversion of resistance. Similarly, the population from Montería, previously reported as resistant [17], was found to be susceptible in the present study. These findings suggest that pirimiphos-methyl could be used as an alternative to malathion in populations that showed susceptibility to this organophosphate.

This study reported moderate–high intensity of resistance for fenitrothion in 78.6% of the analyzed populations, which concurs with previous studies reporting resistance to fenitrothion in the municipalities of San Bernardo del Viento [16] and Montería [17]. Even though fenitrothion has not been used for the past 20 years to control *Ae. aegypti* in the department of Córdoba, it is used to control *Anopheles* spp. through indoor residual spraying (IRS) in areas where malaria outbreaks or epidemics occur [40]. Notably, according to national statistics [6–9], all municipalities in this study contribute to malaria cases, with Tierralta, Puerto Libertador, Montelíbano, and Valencia historically reporting the highest number of cases. The use of fenitrothion to control malaria vectors may affect *Ae. aegypti* populations, leading to selection pressure, and consequently, the development of resistance to the insecticide in these populations.

Taking into account the above and to chronologically describe the use of insecticides in the department of Cordoba, we know that from the 1970s to 2011 temephos was used, which has been replaced by the larval growth regulator diflubenzuron (Dimilin) ​​since 2012. Further, the adulticide pirimiphos-methyl was used between 2016 and 2017, and malathion has been used since 2018 to control *Ae. aegypti*. Additionally, pyrethroids have not been used since 2018, the last pyrethroid used was deltamethrin (K-otrin).

The observed resistance to fenitrothion in all populations, and to pirimiphos-methyl in Montelíbano, Lorica, and Sahagún, could be explained by the action of detoxification enzymes of the α-esterase, β- esterase, or insensitive acetylcholinesterase type because these enzymes were altered in the evaluated populations. However, no statistically significant correlation was found between enzymatic activity and resistance to the evaluated insecticides.

Similar results were reported by Maestre-Serrano et al. [26] in populations from the Colombian Caribbean where the alteration of the enzymes was not associated with insecticide resistance. Likewise, Pareja-Loaiza. [17] also found no association of the enzymatic activity of α-esterases, β-esterases, mixed function oxidases (MFOs), and glutathione-S-transferase (GST) with phenotypic resistance.

In this study, alterations were also observed in the enzyme activity levels of GST, an enzyme related to resistance to the organochlorine DDT and populations resistant to pyrethroids. MFOs showed no alteration in the 14 populations studied. It should be noted that high levels of MFO enzymatic alteration have been previously reported in the Montería population [17], whereas, in the present study, the population did not exhibit altered activity of this enzyme. Furthermore, pNPA esterases were found to be moderately altered in the populations from Valencia and Tierralta; however, there are no reports in Colombia of an association between the alteration of the activity of these enzymes and insecticide resistance.

Results on the activity levels of the detoxification enzymes related to resistance to insecticides used for vector control complement the studies carried out in other departments of the country, such as those conducted in Antioquia, Casanare, Cauca, Huila, Meta, Nariño, Putumayo, Santander, and Valle del Cauca, where MFOs and nonspecific esterases were found to be altered [12, 37, 39, 41, 42]. Furthermore, in the Caribbean Region of Colombia, Maestre-Serrano et al. [26] reported incipient levels of enzymatic alteration for α-esterases and MFOs in Valledupar (Cesar) and Ciénaga (Magdalena), and for GST in Sincelejo (Sucre). Pareja-Loaiza et al. [17] reported enzymatic alterations of α-esterases in Montería (Córdoba); β-esterases in Montería (Córdoba), Barranquilla (Atlántico), Juan de Acosta (Atlántico), and Valledupar (Cesar); MFOs in Juan de Acosta (Atlántico), Montería (Córdoba), and Valledupar (Cesar); and GST in Barranquilla (Atlántico), Cartagena (Bolívar), Juan de Acosta (Atlántico), and Montería (Córdoba).

Detection of mutations in the *Ace-1* gene that encodes the enzyme acetylcholinesterase, the target site of action of organophosphates and carbamates, has become increasingly relevant since the identification of the G119S mutation in a population of *Ae. aegypti* resistant to temephos in the district of Tamil Nadu, India [28]. This is the second study in Colombia and the first in the Caribbean region seeking to identify specific mutations in the *Ae. aegypti Ace-1* gene that may confer resistance to organophosphates used in public health. Nonetheless, in this study, as well as in the one carried out by Grisales et al. [36], the G119S mutation was not detected; however, synonymous mutations were identified that do not reduce, alter, or cause loss of function of the acetylcholinesterase enzyme.

The mutations found here were at positions 1797 (GAC → GAT) and 1983 (ACA → ACT) and did not lead to an amino acid change in the protein sequence. Similarly, a study conducted in Indonesia reported the synonymous mutation T506T, which does not alter the function of the acetylcholinesterase enzyme [43, 44]. Moreover, the Gly12Ser mutation was reported in populations resistant to chlorpyrifos in Mexico [45], whereas the Y455C, I457V, R494M mutations were identified in populations resistant to the neonicotinoid acetamiprid in India [46]. The mutations in *Ace-1* that have been associated with acetylcholinesterase insensitivity in insects are G119S, F290V, and F455W in *Anopheles gambiae*, *Culex pipiens* [47, 48], *Anopheles albimanus* [49], and *Culex tritaeniorhynchus* [50].

In *Ae. aegypti*, there are no reports of the F290V and F455W mutations. Some authors suggest that the absence of these mutations is because of a codon restriction in *Ae. aegypti* that differs in nucleotide sequence from other mosquito species. Furthermore, they indicate that, for the G119S and F455W mutations, two nucleotide mutations are required for these amino acid changes to occur, making it unlikely that these mutations occur spontaneously [36, 44, 51].

Public health entities at the national, departmental, and municipal levels must establish coordination between their vector control programs, as this can contribute to greater program efficiency by sharing information, infrastructure, and human resources [52]. Vector control programs must implement the integrated vector control (IVC) strategy, which aims to improve the efficiency and effectiveness of programs to provide sustainable and adequate control methods that reduce dependence on insecticides and thus protect the population at risk from the most prevalent vector-borne diseases. Among the activities that make up the integrated approach to IVC is the selection of control methods that can be environmental, mechanical, biological, or chemical. However, chemical methods must be implemented taking into account prior information on insecticide resistance, so resistance monitoring must be carried out periodically in the different disease vectors found in the same area [53]; to select the appropriate insecticide and to ensure that the constant use of a specific group of insecticides does not exert indirect selection pressure on other vectors in the same area, generating resistant populations; as in the case of the resistance observed to fenitrothion in *Ae. aegypti*, an insecticide used to control Anopheles mosquitoes in the department of Cordoba.

## Conclusions

Resistance to organophosphates such as fenithrotion and pirimiphos-methyl, as well as susceptibility to malathion and temephos in the studied populations, provide an overview of the current status of susceptibility to organophosphates in the Córdoba department. This information will contribute to improving vector control strategies in this region of Colombia.

## Supplementary Information

**Additional file 1.**

## Data Availability

No datasets were generated or analyzed during the current study. Data supporting the conclusions of this study are in the manuscript and in the additional file. The sequences generated herein were deposited in GenBank under accession numbers PP763441, PP763442, and PP763443.
