# Supplementary material for: Susceptibility to organophosphate insecticides in Aedes aegypti (Diptera: Culicidae) from northern Colombia and associated resistance mechanisms
Source: Parasit Vectors. 2025 Jan 14;18:7. doi: 10.1186/s13071-024-06624-8 (PMC11734419; doi:10.1186/s13071-024-06624-8)
Supplement: Supplementary file 1 — Additional file 1. [file 13071_2024_6624_MOESM1_ESM.docx]

| **Population** | **Temephos (% Mortality)** | | | |
| --- | --- | --- | --- | --- |
|  | **R1** | **R2** | **R3** | **R4** |
| Rockefeller | 100 | 100 | 100 | 100 |
| Cerete | 100 | 100 | 100 | 100 |
| Sahagún | 100 | 100 | 90 | 100 |
| Planeta Rica | 100 | 100 | 100 | 100 |
| San Bernardo del Viento | 100 | 100 | 100 | 100 |
| Lorica | 100 | 100 | 100 | 100 |
| Ayapel | 100 | 100 | 100 | 100 |
| Montelíbano | 100 | 100 | 100 | 100 |
| Tierralta | 100 | 100 | 100 | 100 |
| Pueblo Nuevo | 100 | 100 | 100 | 100 |
| Valencia | 100 | 100 | 100 | 100 |
| Montería | 100 | 100 | 100 | 100 |
| San Andrés de Sotavento | 100 | 100 | 100 | 100 |
| Los Córdoba | 100 | 100 | 100 | 100 |
| Puerto Libertador | 100 | 100 | 100 | 100 |

Additional File 1: Dataset S1. Mortality percentages of the evaluated populations for the organophosphates temephos, fenitrothion, pirimiphos methyl and malathion

| **Population** | **T (min)** | **Fenitrothion (% Mortality)** | | | |
| --- | --- | --- | --- | --- | --- |
|  |  | **R1** | **R2** | **R3** | **R4** |
| Rockefeller | **0** | 0 | 0 | 0 | 0 |
|  | **5** | 0 | 0 | 0 | 0 |
|  | **10** | 0 | 5 | 0 | 0 |
|  | **15** | 12.5 | 36.8 | 15.8 | 6.3 |
|  | **20** | 56.3 | 68.4 | 73.7 | 31.3 |
|  | **25** | 87.5 | 78.9 | 89.5 | 81.3 |
|  | **30** | 100 | 100 | 100 | 100 |
| Cerete | **0** | 0 | 0 | 0 | 0 |
|  | **5** | 0 | 0 | 0 | 0 |
|  | **10** | 0 | 0 | 0 | 0 |
|  | **15** | 0 | 0 | 0 | 0 |
|  | **20** | 0 | 0 | 0 | 0 |
|  | **25** | 0 | 0 | 6.3 | 0 |
|  | **30** | 11.8 | 0 | 18.8 | 0 |
| Sahagún | **0** | 0 | 0 | 0 | 0 |
|  | **5** | 0 | 0 | 0 | 0 |
|  | **10** | 0 | 0 | 0 | 0 |
|  | **15** | 0 | 0 | 0 | 0 |
|  | **20** | 0 | 0 | 0 | 0 |
|  | **25** | 5.6 | 5.9 | 0 | 5.9 |
|  | **30** | 16.7 | 11.8 | 5.9 | 11.8 |
| Planeta Rica | **0** | 0 | 0 | 0 | 0 |
|  | **5** | 0 | 0 | 0 | 0 |
|  | **10** | 0 | 0 | 0 | 0 |
|  | **15** | 0 | 0 | 0 | 0 |
|  | **20** | 0 | 0 | 0 | 0 |
|  | **25** | 0 | 6.3 | 0 | 0 |
|  | **30** | 0 | 18.8 | 0 | 0 |
| San Bernardo del Viento | **0** | 0 | 0 | 0 | 0 |
|  | **5** | 0 | 0 | 0 | 0 |
|  | **10** | 0 | 0 | 0 | 0 |
|  | **15** | 0 | 0 | 0 | 0 |
|  | **20** | 0 | 5 | 0 | 0 |
|  | **25** | 10 | 5 | 0 | 8.7 |
|  | **30** | 30 | 10 | 10 | 30.4 |
| Lorica | **0** | 0 | 0 | 0 | 0 |
|  | **5** | 0 | 0 | 0 | 0 |
|  | **10** | 0 | 0 | 0 | 0 |
|  | **15** | 0 | 0 | 0 | 0 |
|  | **20** | 10 | 5.3 | 0 | 5.3 |
|  | **25** | 20 | 15.8 | 0 | 31.6 |
|  | **30** | 35 | 47.4 | 28.6 | 52.6 |
| Ayapel | **0** | 0 | 0 | 0 | 0 |
|  | **5** | 0 | 0 | 0 | 0 |
|  | **10** | 0 | 0 | 0 | 0 |
|  | **15** | 0 | 0 | 0 | 0 |
|  | **20** | 0 | 0 | 0 | 0 |
|  | **25** | 0 | 0 | 6.3 | 0 |
|  | **30** | 11.8 | 0 | 18.8 | 0 |
| Montelíbano | **0** | 0 | 0 | 0 | 0 |
|  | **5** | 0 | 0 | 0 | 0 |
|  | **10** | 0 | 0 | 0 | 0 |
|  | **15** | 0 | 0 | 0 | 0 |
|  | **20** | 0 | 0 | 0 | 0 |
|  | **25** | 5.6 | 5.9 | 0 | 5.9 |
|  | **30** | 16.7 | 11.8 | 5.9 | 11.8 |
| Tierralta | **0** | 0 | 0 | 0 | 0 |
|  | **5** | 0 | 0 | 0 | 0 |
|  | **10** | 0 | 0 | 0 | 0 |
|  | **15** | 0 | 0 | 0 | 0 |
|  | **20** | 0 | 0 | 0 | 0 |
|  | **25** | 0 | 6.3 | 0 | 0 |
|  | **30** | 0 | 18.8 | 0 | 0 |
| Pueblo Nuevo | **0** | 0 | 0 | 0 | 0 |
|  | **5** | 0 | 0 | 0 | 0 |
|  | **10** | 0 | 0 | 0 | 0 |
|  | **15** | 0 | 0 | 0 | 0 |
|  | **20** | 0 | 5 | 0 | 0 |
|  | **25** | 10 | 5 | 0 | 8.7 |
|  | **30** | 30 | 10 | 10 | 30.4 |
| Valencia | **0** | 0 | 0 | 0 | 0 |
|  | **5** | 0 | 0 | 0 | 0 |
|  | **10** | 0 | 0 | 0 | 0 |
|  | **15** | 0 | 0 | 0 | 0 |
|  | **20** | 10 | 5.3 | 0 | 5.3 |
|  | **25** | 20 | 15.8 | 0 | 31.6 |
|  | **30** | 35 | 47.4 | 28.6 | 52.6 |
| Montería | **0** | 0 | 0 | 0 | 0 |
|  | **5** | 0 | 0 | 0 | 0 |
|  | **10** | 0 | 0 | 0 | 0 |
|  | **15** | 0 | 0 | 0 | 0 |
|  | **20** | 0 | 0 | 0 | 0 |
|  | **25** | 0 | 0 | 0 | 0 |
|  | **30** | 0 | 6 | 29 | 15 |
| San Andrés de Sotavento | **0** | 0 | 0 | 0 | 0 |
|  | **5** | 0 | 0 | 0 | 0 |
|  | **10** | 0 | 0 | 0 | 0 |
|  | **15** | 0 | 0 | 0 | 0 |
|  | **20** | 0 | 0 | 0 | 0 |
|  | **25** | 5 | 4.8 | 4.5 | 5 |
|  | **30** | 5 | 9.5 | 13.6 | 15 |
| Los Córdoba | **0** | 0 | 0 | 0 | 0 |
|  | **5** | 0 | 0 | 0 | 0 |
|  | **10** | 0 | 0 | 0 | 0 |
|  | **15** | 5.3 | 0 | 0 | 0 |
|  | **20** | 15.8 | 5 | 5 | 15 |
|  | **25** | 52.6 | 20 | 30 | 30 |
|  | **30** | 84.2 | 70 | 70 | 65 |
| Puerto Libertador | **0** | 0 | 0 | 0 | 0 |
|  | **5** | 0 | 0 | 0 | 0 |
|  | **10** | 0 | 0 | 0 | 0 |
|  | **15** | 0 | 0 | 0 | 0 |
|  | **20** | 0 | 0 | 0 | 0 |
|  | **25** | 0 | 5.6 | 0 | 0 |
|  | **30** | 5.3 | 11.1 | 5.6 | 5.9 |

| **Population** | **T (min)** | **Pirimiphos-metyl (% Mortality)** | | | |
| --- | --- | --- | --- | --- | --- |
|  |  | **R1** | **R2** | **R3** | **R4** |
| Rockefeller | **0** | 0 | 0 | 0 | 0 |
|  | **5** | 0 | 0 | 0 | 0 |
|  | **10** | 0 | 0 | 0 | 0 |
|  | **15** | 20 | 5.9 | 17.6 | 10 |
|  | **20** | 60 | 47.1 | 58.8 | 45 |
|  | **25** | 93.3 | 88.2 | 88.2 | 85 |
|  | **30** | 93.3 | 88.2 | 100 | 95 |
|  | **35** | 100 | 94.1 | 100 | 95 |
|  | **40** | 100 | 100 | 100 | 95 |
|  | **45** | 100 | 100 | 100 | 100 |
| Cerete | **0** | 0 | 0 | 0 | 0 |
|  | **5** | 4.3 | 0 | 0 | 5.9 |
|  | **10** | 4.3 | 0 | 0 | 5.9 |
|  | **15** | 34.8 | 26,0 | 29,0 | 35.3 |
|  | **20** | 87,0 | 95,0 | 67,0 | 82.4 |
|  | **25** | 100 | 95,0 | 92,0 | 94.1 |
|  | **30** | 100 | 100 | 100 | 100 |
|  | **35** | 100 | 100 | 100 | 100 |
|  | **40** | 100 | 100 | 100 | 100 |
|  | **45** | 100 | 100 | 100 | 100 |
| Sahagún | **0** | 0 | 0 | 0 | 0 |
|  | **5** | 0 | 0 | 0 | 0 |
|  | **10** | 0 | 0 | 0 | 0 |
|  | **15** | 0 | 0 | 0 | 0 |
|  | **20** | 6.3 | 5.6 | 5.9 | 13.3 |
|  | **25** | 6.3 | 16.7 | 41.2 | 40 |
|  | **30** | 43.8 | 38.9 | 29.4 | 80 |
|  | **35** | 68.8 | 72.2 | 88.2 | 93.3 |
|  | **40** | 87.5 | 83.3 | 100 | 100 |
|  | **45** | 93.8 | 88.9 | 100 | 100 |
| Planeta Rica | **0** | 0 | 0 | 0 | 0 |
|  | **5** | 0 | 0 | 0 | 0 |
|  | **10** | 0 | 0 | 0 | 0 |
|  | **15** | 0 | 0 | 0 | 15,0 |
|  | **20** | 5 | 11 | 22 | 45 |
|  | **25** | 50 | 47 | 44 | 80 |
|  | **30** | 85 | 84 | 83 | 95 |
|  | **35** | 90 | 95 | 94 | 100 |
|  | **40** | 100 | 100 | 100 | 100 |
|  | **45** | 100 | 100 | 100 | 100 |
| San Bernardo del Viento | **0** | 0 | 0 | 0 | 0 |
|  | **5** | 0 | 0 | 0 | 0 |
|  | **10** | 0 | 0 | 0 | 0 |
|  | **15** | 0 | 0 | 0 | 0 |
|  | **20** | 5.3 | 11 | 5 | 5.6 |
|  | **25** | 10.5 | 16 | 11 | 11.1 |
|  | **30** | 26.3 | 32 | 32 | 27.8 |
|  | **35** | 57.9 | 63 | 58 | 55.6 |
|  | **40** | 89.5 | 95 | 89 | 88.9 |
|  | **45** | 100 | 100 | 95 | 100 |
| Lorica | **0** | 0 | 0 | 0 | 0 |
|  | **5** | 0 | 0 | 0 | 0 |
|  | **10** | 0 | 0 | 0 | 0 |
|  | **15** | 0 | 0 | 0 | 0 |
|  | **20** | 0 | 0 | 4.8 | 4.5 |
|  | **25** | 13 | 10 | 14.3 | 9.1 |
|  | **30** | 26.1 | 25 | 23.8 | 18.2 |
|  | **35** | 43.5 | 45 | 47.6 | 40.9 |
|  | **40** | 82.6 | 75 | 71.4 | 77.3 |
|  | **45** | 100 | 90 | 81 | 86.4 |
| Ayapel | **0** | 0 | 0 | 0 | 0 |
|  | **5** | 0 | 0 | 0 | 4.8 |
|  | **10** | 0 | 0 | 0 | 4.8 |
|  | **15** | 0 | 10 | 5.3 | 9.5 |
|  | **20** | 20 | 30 | 52.6 | 52.4 |
|  | **25** | 75 | 85 | 84.2 | 76.2 |
|  | **30** | 95 | 95 | 89.5 | 90.5 |
|  | **35** | 100 | 100 | 100 | 100 |
|  | **40** | 100 | 100,0 | 100 | 100 |
|  | **45** | 100 | 100 | 100 | 100 |
| Montelíbano | **0** | 0 | 0 | 0 | 0 |
|  | **5** | 0 | 0 | 0 | 0 |
|  | **10** | 0 | 0 | 0 | 0 |
|  | **15** | 0 | 0 | 0 | 0 |
|  | **20** | 0 | 0 | 0 | 0 |
|  | **25** | 5.6 | 0 | 0 | 0 |
|  | **30** | 5.6 | 4.8 | 4.8 | 14.3 |
|  | **35** | 22.2 | 28.6 | 33.3 | 28.6 |
|  | **40** | 50 | 42.9 | 52.4 | 47.6 |
|  | **45** | 77.8 | 61.9 | 85.7 | 76.2 |
| Tierralta | **0** | 0 | 0 | 0 | 0 |
|  | **5** | 0 | 0 | 0 | 0 |
|  | **10** | 0 | 0 | 0 | 0 |
|  | **15** | 0 | 0 | 0 | 0 |
|  | **20** | 0 | 0 | 0 | 0 |
|  | **25** | 5.6 | 0 | 0 | 0 |
|  | **30** | 5.6 | 4.8 | 4.8 | 14.3 |
|  | **35** | 22.2 | 28.6 | 33.3 | 28.6 |
|  | **40** | 50 | 42.9 | 52.4 | 47.6 |
|  | **45** | 77.8 | 61.9 | 85.7 | 76.2 |
| Pueblo Nuevo | **0** | 0 | 0 | 0 | 0 |
|  | **5** | 0 | 0 | 0 | 0 |
|  | **10** | 0 | 0 | 0 | 0 |
|  | **15** | 0 | 0 | 0 | 0 |
|  | **20** | 5 | 15 | 10 | 10.5 |
|  | **25** | 65 | 85 | 55 | 47.4 |
|  | **30** | 95 | 100 | 90 | 78.9 |
|  | **35** | 100 | 100 | 95 | 84.2 |
|  | **40** | 100 | 100 | 100 | 100 |
|  | **45** | 100 | 100 | 100 | 100 |
| Valencia | **0** | 0 | 0 | 0 | 0 |
|  | **5** | 0 | 0 | 0 | 0 |
|  | **10** | 0 | 0 | 0 | 0 |
|  | **15** | 5.3 | 0 | 8.7 | 0 |
|  | **20** | 31.6 | 14.3 | 34.8 | 9.1 |
|  | **25** | 52.6 | 47.6 | 65.2 | 54.5 |
|  | **30** | 89.5 | 95.2 | 91.3 | 95.5 |
|  | **35** | 100 | 95.2 | 91.3 | 100 |
|  | **40** | 100 | 95.2 | 95.7 | 100 |
|  | **45** | 100 | 100 | 100 | 100 |
| Montería | **0** | 0 | 0 | 0 | 0 |
|  | **5** | 0 | 0 | 0 | 0 |
|  | **10** | 0 | 0 | 0 | 0 |
|  | **15** | 5.6 | 4.8 | 0 | 5.3 |
|  | **20** | 16.7 | 19 | 15 | 31.6 |
|  | **25** | 55.6 | 42.9 | 55 | 73.7 |
|  | **30** | 88.9 | 85.7 | 95 | 94.7 |
|  | **35** | 100 | 100 | 100 | 100 |
|  | **40** | 100 | 100 | 100 | 100 |
|  | **45** | 100 | 100 | 100 | 100 |
| San Andres de Sotavento | **0** | 0 | 0 | 0 | 0 |
|  | **5** | 0 | 0 | 0 | 0 |
|  | **10** | 0 | 0 | 0 | 0 |
|  | **15** | 10 | 0 | 0 | 11.8 |
|  | **20** | 50 | 11.8 | 15.8 | 70.6 |
|  | **25** | 85 | 29.4 | 31.6 | 88.2 |
|  | **30** | 95 | 70.6 | 68.4 | 100 |
|  | **35** | 100 | 94.1 | 89.5 | 100 |
|  | **40** | 100 | 100 | 94.7 | 100 |
|  | **45** | 100 | 100 | 100 | 100 |
| Los Córdoba | **0** | 0 | 0 | 0 | 0 |
|  | **5** | 0 | 0 | 0 | 0 |
|  | **10** | 0 | 0 | 0 | 0 |
|  | **15** | 4.3 | 0 | 8.3 | 13.6 |
|  | **20** | 69.6 | 40.9 | 58.3 | 40.9 |
|  | **25** | 95.7 | 81.8 | 87.5 | 81.8 |
|  | **30** | 100 | 86.4 | 91.7 | 81.8 |
|  | **35** | 100 | 90.9 | 95.8 | 90.9 |
|  | **40** | 100 | 95.5 | 95.8 | 95.5 |
|  | **45** | 100 | 95.5 | 100 | 100 |
| Puerto Libertador | **0** | 0 | 0 | 0 | 0 |
|  | **5** | 0 | 0 | 0 | 0 |
|  | **10** | 0 | 0 | 0 | 0 |
|  | **15** | 0 | 0 | 0 | 0 |
|  | **20** | 33.3 | 25 | 37.5 | 15 |
|  | **25** | 66.7 | 75 | 75 | 50 |
|  | **30** | 80 | 93.8 | 100 | 80 |
|  | **35** | 100 | 93.8 | 100 | 100 |
|  | **40** | 100 | 100 | 100 | 100 |
|  | **45** | 100 | 100 | 100 | 100 |

| **Population** | **T (min)** | **Malathion (% Mortality)** | | | |
| --- | --- | --- | --- | --- | --- |
|  |  | **R1** | **R2** | **R3** | **R4** |
| Rockefeller | **0** | 0 | 0 | 0 | 0 |
|  | **5** | 0 | 0 | 0 | 0 |
|  | **10** | 20 | 31.3 | 19 | 23.5 |
|  | **15** | 80 | 75,0 | 85.7 | 76.5 |
|  | **20** | 100 | 93.8 | 95.2 | 100 |
|  | **25** | 100 | 100 | 100 | 100 |
|  | **30** | 100 | 100 | 100 | 100 |
| Cerete | **0** | 0 | 0 | 0 | 0 |
|  | **5** | 0 | 0 | 0 | 0 |
|  | **10** | 4.8 | 5 | 5 | 4.8 |
|  | **15** | 90.5 | 90 | 90 | 90.5 |
|  | **20** | 95.2 | 100 | 100 | 95.2 |
|  | **25** | 100 | 100 | 100 | 95.2 |
|  | **30** | 100 | 100 | 100 | 100 |
| Sahagún | **0** | 0 | 0 | 0 | 0 |
|  | **5** | 0 | 0 | 0 | 0 |
|  | **10** | 0 | 0 | 0 | 0 |
|  | **15** | 21.1 | 17 | 26 | 47.1 |
|  | **20** | 89.5 | 83 | 95 | 100 |
|  | **25** | 100 | 94 | 100 | 100 |
|  | **30** | 100 | 100 | 100 | 100 |
| Planeta Rica | **0** | 0 | 0 | 0 | 0 |
|  | **5** | 0 | 0 | 0 | 0 |
|  | **10** | 0 | 0 | 0 | 0 |
|  | **15** | 10 | 9 | 6 | 5.6 |
|  | **20** | 60 | 41 | 44 | 44.4 |
|  | **25** | 100 | 95 | 89 | 88.9 |
|  | **30** | 100 | 100 | 100 | 100 |
| San Bernardo del Viento | **0** | 0 | 0 | 0 | 0 |
|  | **5** | 0 | 0 | 0 | 0 |
|  | **10** | 0 | 5 | 0 | 5.3 |
|  | **15** | 5.9 | 26 | 28 | 26.3 |
|  | **20** | 35.3 | 53 | 72 | 52.6 |
|  | **25** | 94.1 | 95 | 100 | 94.7 |
|  | **30** | 100 | 100 | 100 | 100 |
| Lorica | **0** | 0 | 0 | 0 | 0 |
|  | **5** | 0 | 0 | 0 | 0 |
|  | **10** | 0 | 0 | 0 | 0 |
|  | **15** | 17.6 | 11 | 16 | 11.1 |
|  | **20** | 52.9 | 39 | 42 | 33.3 |
|  | **25** | 94.1 | 89 | 89 | 88.9 |
|  | **30** | 100 | 100 | 100 | 100 |
| Ayapel | **0** | 0 | 0 | 0 | 0 |
|  | **5** | 0 | 0 | 0 | 0 |
|  | **10** | 5.3 | 11 | 12 | 13.3 |
|  | **15** | 84.2 | 89 | 88 | 86.7 |
|  | **20** | 100 | 100 | 100 | 100 |
|  | **25** | 100 | 100 | 100 | 100 |
|  | **30** | 100 | 100 | 100 | 100 |
| Montelíbano | **0** | 0 | 0 | 0 | 0 |
|  | **5** | 0 | 0 | 0 | 0 |
|  | **10** | 0 | 0 | 0 | 4.8 |
|  | **15** | 14.3 | 25 | 19 | 19 |
|  | **20** | 90.5 | 90 | 86 | 85.7 |
|  | **25** | 95.2 | 100 | 100 | 95.2 |
|  | **30** | 100 | 100 | 100 | 100 |
| Tierralta | **0** | 0 | 0 | 0 | 0 |
|  | **5** | 0 | 0 | 0 | 0 |
|  | **10** | 0 | 0 | 0 | 0 |
|  | **15** | 10.5 | 35.3 | 40 | 37.5 |
|  | **20** | 52.6 | 70.6 | 65 | 81.3 |
|  | **25** | 94.7 | 94.1 | 95 | 93.8 |
|  | **30** | 100 | 100 | 100 | 100 |
| Pueblo Nuevo | **0** | 0 | 0 | 0 | 0 |
|  | **5** | 0 | 0 | 0 | 0 |
|  | **10** | 0 | 0 | 0 | 0 |
|  | **15** | 10.5 | 0 | 6 | 5.6 |
|  | **20** | 47.4 | 16 | 25 | 27.8 |
|  | **25** | 94.7 | 68 | 69 | 66.7 |
|  | **30** | 100 | 100 | 100 | 100 |
| Valencia | **0** | 0 | 0 | 0 | 0 |
|  | **5** | 0 | 0 | 0 | 0 |
|  | **10** | 5.9 | 6 | 0 | 5.9 |
|  | **15** | 17.6 | 47 | 19 | 29.4 |
|  | **20** | 64.7 | 71 | 71 | 82.4 |
|  | **25** | 94.1 | 94 | 95 | 94.1 |
|  | **30** | 100 | 100 | 100 | 100 |
| Montería | **0** | 0 | 0 | 0 | 0 |
|  | **5** | 0 | 0 | 0 | 0 |
|  | **10** | 0 | 6 | 0 | 0 |
|  | **15** | 37.5 | 25 | 30 | 26.7 |
|  | **20** | 100 | 94 | 100 | 80 |
|  | **25** | 100 | 100 | 100 | 100 |
|  | **30** | 100 | 100 | 100 | 100 |
| San Andrés de Sotavento | **0** | 0 | 0 | 0 | 0 |
|  | **5** | 0 | 0 | 0 | 0 |
|  | **10** | 0 | 0 | 13 | 0 |
|  | **15** | 11.1 | 11 | 31 | 5 |
|  | **20** | 55.6 | 61 | 75 | 40 |
|  | **25** | 88.9 | 94 | 100 | 100 |
|  | **30** | 100 | 100 | 100 | 100 |
| Los Córdoba | **0** | 0 | 0 | 0 | 0 |
|  | **5** | 0 | 0 | 0 | 0 |
|  | **10** | 0 | 0 | 0 | 5.3 |
|  | **15** | 16.7 | 22 | 22 | 26.3 |
|  | **20** | 72.2 | 83 | 67 | 68.4 |
|  | **25** | 100 | 94 | 89 | 100 |
|  | **30** | 100 | 100 | 100 | 100 |
| Puerto Libertador | **0** | 0 | 0 | 0 | 0 |
|  | **5** | 0 | 0 | 0 | 0 |
|  | **10** | 0 | 0 | 0 | 5.6 |
|  | **15** | 25 | 37 | 20 | 27.8 |
|  | **20** | 80 | 89 | 85 | 94.4 |
|  | **25** | 95 | 100 | 100 | 100 |
|  | **30** | 100 | 100 | 100 | 100 |
